# Numerical Prediction of Two-Phase Flow through a Tube Bundle Based on Reduced-Order Model and a Void Fraction Correlation

**DOI:** 10.3390/e23101355

**Published:** 2021-10-16

**Authors:** Claire Dubot, Cyrille Allery, Vincent Melot, Claudine Béghein, Mourad Oulghelou, Clément Bonneau

**Affiliations:** 1 LaSIE, UMR-7356-CNRS, La Rochelle Université, Avenue Michel Crépeau, 17042 La Rochelle, France; cyrille.allery@univ-lr.fr (C.A.); Claudine.Beghein@univ-lr.fr (C.B.); 2 Naval Group, Rue du Bac, 44620 La Montagne, France; Vincent.Melot@naval-group.com (V.M.); clement.bonneau@naval-group.com (C.B.); 3 LAMPA, ENSAM Paris Tech, 2 Boulevard de Ronceray, 49035 Angers, France; mourad.oulghelou@ensam.eu

**Keywords:** steam generator, void fraction, mixture model, porous media approach, reduced-order model, Proper Orthogonal Decomposition (POD)

## Abstract

Predicting the void fraction of a two-phase flow outside of tubes is essential to evaluate the thermohydraulic behaviour in steam generators. Indeed, it determines two-phase mixture properties and affects two-phase mixture velocity, which enable evaluating the pressure drop of the system. The two-fluid model for the numerical simulation of two-phase flows requires interaction laws between phases which are not known and/or reliable for a flow within a tube bundle. Therefore, the mixture model, for which it is easier to implement suitable correlations for tube bundles, is used. Indeed, by expressing the relative velocity as a function of slip, the void fraction model of Feenstra et al. and Hibiki et al. developed for upward cross-flow through horizontal tube bundles is introduced and compared. With the method suggested in this paper, the physical phenomena that occur in tube bundles are taken into consideration. Moreover, the tube bundle is modelled using a porous media approach where the Darcy–Forchheimer term is usually defined by correlations found in the literature. However, for some tube bundle geometries, these correlations are not available. The second goal of the paper is to quickly compute, in quasi-real-time, this term by a non-intrusive parametric reduced model based on Proper Orthogonal Decomposition. This method, named Bi-CITSGM (Bi-Calibrated Interpolation on the Tangent Subspace of the Grassmann Manifold), consists in interpolating the spatial and temporal bases by ITSGM (Interpolation on the Tangent Subspace of the Grassmann Manifold) in order to define the solution for a new parameter. The two developed methods are validated based on the experimental results obtained by Dowlati et al. for a two-phase cross-flow through a horizontal tube bundle.

## 1. Introduction

Steam generators are heat exchangers used especially in nuclear propulsion. Water, heated by the reactor core, flows through a tube bundle, which is a closed circuit called the primary circuit. The heat of the primary fluid is diffused by conduction through metallic tube walls to the water, which flows outside the tubes. Water in the secondary circuit, also called the secondary fluid, enters in a liquid state and becomes a two-phase mixture of steam and water as heat transfer occurs along the heat exchanger. The steam is then used to generate electricity using rotating turbines.

A three-dimensional thermo-hydraulic analysis is essential to predict the performance of heat exchangers and their correct design, especially taking into account that the tube bundle, where there may be thousands of tubes, would require unacceptable computational cost and time. Therefore, the whole code cited in this paper models the tube bundle as a porous medium. In the case of two-phase flows, the void fraction is the key parameter to characterize the flow. Indeed, it enables calculating the mixture density, the mixture viscosity, and the mixture velocity. Consequently, it plays an important role for computing pressure drops and heat and mass transfers.

Since the 1980s, thermal-hydraulic codes have been developed to understand the physical phenomena involved. One of the first codes, THIRST [1], was developed to compute three-dimensional, two-phase, and steady flow in steam generators. The two-phase flow was solved by the homogeneous two-phase model, and the phase velocities were assumed to be equal, but this does not reflect what is really going on. To take into account the slip between phases, Navier–Stokes equations were solved for the mixture of the secondary fluid. The THYC code [2] gives the relative velocity thanks to a correlation using the drift flux model of Zuber and Findlay [3]. This model is based on the determination of drift-flux parameters for which there are many different empirical correlations [4,5,6,7,8]. Stevanovic et al. [9] used the two-fluid model to predict the thermal-hydraulic behavior in horizontal tube bundles. Navier–Stokes equations were solved for each phase. The accurate definition of the interfacial drag force, comprising a drag coefficient correlation, is important in order to predict the void fraction distribution. In their paper, the original drag correlation of Ishii and Zuber [10] was multiplied by 0.4. They validated this modification of the correlation with their experimental data. Nevertheless, most drag coefficient laws in the literature [11,12,13,14,15], including Ishii and Zuber’s correlation, are made for different two-phase flow regimes (bubbly, slug, stratified, annular, or spray flow) inside a tube and not in tube bundles. In this study, this problem is tackled by using the mixture model, which is a simplified two-phase model where Navier–Stokes equations are solved for the mixture. The developed method involved formulating the relative velocity as a function of slip and then implementing a specific void fraction model for tube bundles derived from the literature.

The slip ratio is defined as the ratio between gas phase velocity and liquid phase velocity. Feenstra et al. [16] developed a slip ratio model based on their R-11 data for upward two-phase cross-flow through horizontal tube bundles. They identified the important variables that affected slip, and the application of the Buckingham pi theorem enabled them to reduce the slip ratio as a function of two dimensionless numbers, namely the Richardson number and the Capillary number. They demonstrated that it fitted well with experimental void fraction data in R-11 and air–water mixtures for a wide range of mass fluxes, qualities and pitch-diameter ratios. This model is not explicit for void fraction; indeed an iterative process is necessary to compute the void fraction. Likewise, Hibiki et al. [17] used the slip ratio correlation of Smith [18] to develop a correlation for the entrainment factor dependent on the tube’s bundle arrangement. The entrainment factor is correlated with a dominant parameter such as nondimensional mass flux based on experimental data coming from various flow configurations and tube bundle arrangements. The developed correlation agrees both with parallel and crossflow in the tube bundle.

Moreover, the porous media approach implies adding a momentum sink to the governing momentum equation. This source term is defined by the Darcy–Forchheimer law [19,20]. A widely used and easy method is the use of correlations coming from the literature, such as Zukauskas et al.’s correlation [21] for a transverse flow to a tube bundle or Rhodes and Carlucci’s correlation [22] for a parallel flow. These correlations result from experiments, it seems to be the most accurate method to determine the law but it is valid for a given design and it is expensive and time-consuming. However, for new, i.e., non-standard, tube geometry, these correlations are not available, and solving a reduced-order model to define them is suggested. From some CFD calculations of flow through a Representative Elementary Volume (REV) of the tube bundle, pressure and velocity fields were decomposed by POD (Proper Orthogonal Decomposition). Then, the non-intrusive reduced model method, Bi-CITSGM (Bi-Calibrated Interpolation on the Tangent Subspace of the Grassmann Manifold) [23,24], was applied in order to determine the solution for a new parameter. In this method, spatial and temporal POD sampling bases are interpolated by ITSGM (Interpolation on the Tangent Subspace of the Grassmann Manifold) [25] and the temporal eigenvalues by usual methods such as Lagrange, IDW (Inverse Distance Weighting), or RBF (Radial Basis Function). Then, the reduced-order model was used to compute the source term of the porous media approach applied to the flow through a tube bundle.

**Figure 1:**
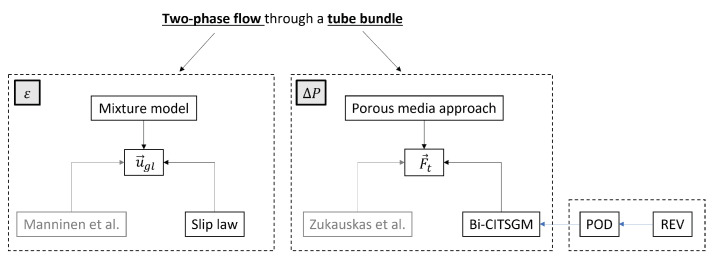
Summary of the suggested approach. On the left side, the mixture model is modified by Feenstra’s correlation to compute the void fraction. On the right side, the Bi-CITSGM method applied to a REV is introduced in the porous media approach to define the pressure drop of the tube bundle.

This work was validated with the experimental results of Dowlati et al. [26] which is a two-phase cross-flow through a horizontal tube bundle. The summary of the developed methodology is presented in Figure 1. First, the mixture model was used, and we rewrote the slip velocity, ${\vec{u}}_{gl}$, as a function of the slip in order to implement a specific void fraction model. The implementation of Feenstra’s correlation and Hibiki’s correlation were compared to the usual formulation of Manninen et al. [27]. Moreover, the tube bundle was modelled by a porous medium. The ability to use a reduced-order model to determine the momentum sink term ${\vec{F}}_{t}$ was studied and compared to the usual correlation of Zukauskas et al. [21].

The remainder of the paper is organized as follows. In Section 2, the governing equations used to model the two-phase flow through a tube bundle by a porous media approach are presented. The rewriting of the relative velocity and the Darcy–Forchheimer term are also detailed. Section 3 deals with the methodology of the reduced-order model on the REV to compute the Forchheimer term. The ITSGM method and the non-intrusive approach Bi-CITSGM are reviewed. Section 4 validates the application of the proposed reformulation of the relative velocity, and Section 5 confirms the use of a ROM to determine the momentum sink term. Finally, conclusions are drawn.

## 2. Cross-Flow through a Horizontal Tube Bundle

**Figure 2:**
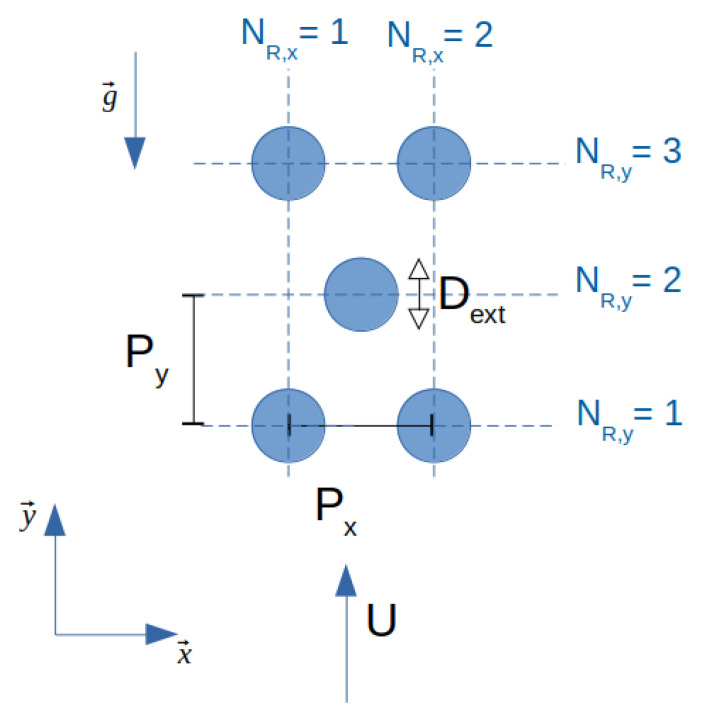
Geometric definitions of the tube bundle.

This study focuses on the simulation of the thermal-hydraulic behaviour of a two-phase adiabatic crossflow through a horizontal tube bundle. The tube bundle is modelled by a porous medium that involves solving the conservation equations of mass and momentum and the turbulence model using the superficial velocity porous formulation. The flow is considered two-dimensional and only transverse to the tube bundle. All geometric notations pertaining to the tube bundle are illustrated in Figure 2.

### 2.1. Governing Equations of the Two-Phase Flow

In order to simulate the adiabatic two-phase flow, the mixture model is used. Only two phases are considered: the liquid phase *l* and the gas phase *g*. The mixture model allows the phases to be interpenetrating and to move at different velocities using the concept of slip velocities. The continuity equation for the mixture is

(1) $$\frac{\partial }{\partial t}\left(\rho_{b}\right)+\nabla \cdot \left(\rho_{b}{\vec{u}}_{b}\right)=0$$

where ${\vec{u}}_{b}$ is the mass-averaged velocity defined by

(2) $${\vec{u}}_{b}=\frac{\alpha_{g}\rho_{g}{\vec{u}}_{g}+\alpha_{l}\rho_{l}{\vec{u}}_{l}}{\rho_{b}}.$$

$\alpha_{k}$ is the volume fraction of phase *k*, and $\rho_{b}$ is the mixture density, defined by

(3) $$\rho_{b}=\alpha_{g}\rho_{g}+\alpha_{l}\rho_{l}.$$

The momentum equation for the mixture is obtained by summing the individual momentum equations of all phases. It takes the following form

(4) $$\begin{array}{c} \frac{\partial }{\partial t}\left(\rho_{b}{\vec{u}}_{b}\right)+\nabla \cdot \left(\rho_{b}{\vec{u}}_{b}⊗{\vec{u}}_{b}\right)=-\nabla p+\nabla \cdot \left[\mu_{b}\left(\nabla {\vec{u}}_{b}+\nabla {\vec{u}}_{b}^{T}\right)\right]+\;\rho_{b}\vec{g}\\ -\nabla \cdot \left(\alpha_{g}\rho_{g}{\vec{u}}_{dr,g}{\vec{u}}_{dr,g}+\alpha_{l}\rho_{l}{\vec{u}}_{dr,l}{\vec{u}}_{dr,l}\right)+{\vec{F}}_{t}. \end{array}$$

$\mu_{b}$ is the mixture viscosity such as

(5) $$\mu_{b}=\alpha_{g}\mu_{g}+\alpha_{l}\mu_{l}$$

and $\vec{g}=-g\vec{y}$ with $g=9.81\;m/s^{2}$. The hydraulic resistance of tubes on the fluid is taken into account by the Darcy–Forchheimer term ${\vec{F}}_{t}$. This is a source term due to the use of the porous media approach and it is defined in the following Section 2.3. ${\vec{u}}_{dr,k}$ is the drift velocity of the phase *k* defined by

(6) $${\vec{u}}_{dr,k}={\vec{u}}_{k}-{\vec{u}}_{b}.$$

Moreover, the volume fraction equation for phase *g* is built from the continuity equation for the gas phase, using the definition of the drift velocity (Equation (6)) to eliminate the phase velocity as:

(7) $$\frac{\partial }{\partial t}\left(\alpha_{g}\rho_{g}\right)+\nabla \;\cdot \left(\alpha_{g}\rho_{g}{\vec{u}}_{b}\right)=-\nabla \cdot \left(\alpha_{g}\rho_{g}{\vec{u}}_{dr,g}\right).$$

The drift velocity is related to the relative (or slip) velocity according to:

(8) $${\vec{u}}_{dr,g}={\vec{u}}_{gl}\left(1-\frac{\alpha_{g}\rho_{g}}{\rho_{b}}\right)\;and\;{\vec{u}}_{dr,l}={\vec{u}}_{gl}\left(\frac{\alpha_{l}\rho_{l}}{\rho_{b}}-1\right).$$

The most used algebraic slip formulation is the Manninen model [27]. With this formulation, the slip velocity is given by

(9) $${\vec{u}}_{gl}=\frac{\tau_{g}}{f_{drag}}\frac{\left(\rho_{g}-\rho_{b}\right)}{\rho_{g}}\;\vec{a}$$

where $\tau_{g}$ is the particle relaxation time,

(10) $$\tau_{g}=\frac{\rho_{g}d_{g}^{2}}{18\mu_{g}}$$

$d_{g}$ is the gas particle diameter and $\vec{a}$ is the acceleration.

(11) $$\vec{a}=\vec{g}-\left({\vec{u}}_{b}\cdot \nabla \right){\vec{u}}_{b}-\frac{\partial {\vec{u}}_{b}}{\partial t}$$

The drag force $f_{drag}$ is obtained with the model of Schiller and Naumann [11]. Commonly used, it is expressed as a function of the drag coefficient $C_{D}$ and the relative Reynolds number:

(12) $$f_{drag}=\frac{C_{D}Re}{24}\;and\;Re=\frac{\rho_{l}∥{\vec{u}}_{g}-{\vec{u}}_{l}∥d_{g}}{\mu_{l}}$$

This slip velocity formulation is not suitable for a two-phase flow in tube bundles because it is not designed for such a configuration and does not take into account the associated physical phenomena. In the next subsection, a formulation more adequate for the tube bundle configuration is suggested.

### 2.2. Rewriting of the Slip Velocity

The void fraction depends on the slip ratio *S* and quality *x* according to

(13) $$\varepsilon =\frac{1}{1+S\frac{\rho_{g}}{\rho_{l}}\frac{1-x}{x}}$$

Here, the slip velocity is reformulated as a function of slip in order to introduce a slip ratio model adapted to a two-phase through a tube bundle. The slip velocity is the velocity difference between the gas phase and the liquid phase.

(14) $${\vec{u}}_{gl}={\vec{u}}_{g}-{\vec{u}}_{l}$$

The velocity components in Equation (14) can be written as

(15) $$\left\{\begin{array}{c} u_{gl,x}=u_{l,x}\left(\frac{u_{g,x}}{u_{l,x}}-1\right)=u_{l,x}\left(S_{x}-1\right)\\ u_{gl,y}=u_{l,y}\left(\frac{u_{g,y}}{u_{l,y}}-1\right)=u_{l,y}\left(S_{y}-1\right) \end{array}\right.$$

$u_{l,x}$ (resp. $u_{l,y}$) is the liquid velocity in the $\vec{x}$ (resp. $\vec{y}$) direction. The same notation is used for the gas velocity and the slip velocity.

For an upward cross-flow to the tube bundle in the y direction, it can be assumed that the velocity ratio $S_{x}$ is equal to 1 and that $S_{y}$ is defined by a correlation coming from the literature. Finally, the drift velocity is written as

(16) $$\left\{\begin{array}{c} u_{dr,g,x}=u_{l,x}\left(S_{x}-1\right)\left(1-\frac{\alpha_{g}\rho_{g}}{\rho_{b}}\right)\\ u_{dr,g,y}=u_{l,y}\left(S_{y}-1\right)\left(1-\frac{\alpha_{g}\rho_{g}}{\rho_{b}}\right) \end{array}\right.$$

The proposed approach needs to solve Equations (1), (4) and (7) with the modified definition of the drift velocity defined by Equation (16).

#### 2.2.1. Hibiki’s Correlation (2017)

Smith [18] gives the slip ratio valid for all void fraction ranges and for vertical, inclined, and horizontal flows in a channel [28,29] as

(17) $$S=e+\left(1-e\right){\left(\frac{\frac{\rho_{l}}{\rho_{g}}+\left(\frac{1-x}{x}\right)e}{1+\left(\frac{1-x}{x}\right)e}\right)}^{0.5}$$

where *e* is the ratio of the mass of liquid droplets entrained in the gas core to the total mass of liquid. In order to implement a void fraction correlation in a steam generator thermal-hydraulic code, Hibiki et al. defined this parameter for staggered tube bundles like Dowlati et al. as

(18) $$e=min(0.0637N_{\dot{m},p}^{0.571},1).$$

$N_{\dot{m},p}={\dot{m}}_{p}/\rho_{g}j_{g,crit}$ where the critical superficial gas velocity is

(19) $$j_{g,crit}={\left(\frac{\Delta \rho g\sigma }{\rho_{g}^{2}}\right)}^{0.25}{\left(\frac{\mu_{l}}{{\left(\rho_{l}\sigma \sqrt{\frac{\sigma }{g\Delta \rho }}\right)}^{0.5}}\right)}^{-0.2}.$$

where σ is the surface tension. ${\dot{m}}_{p}$ is the pitch mass flux, which represents the mixture velocity between two tubes $∥{\vec{u}}_{b,p}∥$ multiplied by the mixture density.

(20) $${\dot{m}}_{p}=\alpha_{g}\rho_{g}∥{\vec{u}}_{g,p}∥+\alpha_{l}\rho_{l}∥{\vec{u}}_{l,p}∥\;\mathrm{where}\;∥{\vec{u}}_{k,p}∥=∥{\vec{u}}_{k}∥\frac{P}{P-D_{ext}}\;\mathrm{for}\;k=\left\{l,g\right\}$$

#### 2.2.2. Feenstra’s Correlation (2000)

Feenstra et al. [16] defined the slip ratio as

(21) $$S=1+25.7\sqrt{Ri*Cap}{\left(\frac{P}{D_{ext}}\right)}^{-1}$$

$D_{ext}$ is the outer diameter of the tubes, and *P* is the pitch, illustrated in Figure 2. The Richardson number is the ratio between the buoyancy force and the inertia force:

(22) $$Ri=\frac{{\left(\rho_{g}-\rho_{l}\right)}^{2}g\left(P-D_{ext}\right)}{{\dot{m_{p}}}^{2}}$$

The Capillary number is the ratio between the viscous force and the surface tension force:

(23) $$Cap=\frac{\mu_{l}∥{\vec{u}}_{g,p}∥}{\sigma }$$

The gas phase velocity is based on the resulting void fraction:

(24) $$∥{\vec{u}}_{g,p}∥=\frac{x{\dot{m}}_{p}}{\varepsilon \rho_{g}}$$

### 2.3. Definition of the Darcy–Forchheimer Term

The source term ${\vec{F}}_{t}=F_{t,x}\vec{x}+F_{t,y}\vec{y}$ is added to the momentum equation because tube bundles are represented by a porous medium and is usually written as:

(25) $$\left\{\begin{array}{cc} F_{t,x} & =-\left(D_{xx}\mu_{b}u_{b,x}+K_{xx}\frac{1}{2}\rho_{b}∥{\vec{u}}_{b}∥u_{b,x}\right)\\ F_{t,y} & =-\left(D_{yy}\mu_{b}u_{b,y}+K_{yy}\frac{1}{2}\rho_{b}∥{\vec{u}}_{b}∥u_{b,y}\right) \end{array}\right.$$

This term is composed of a viscous loss term and an inertial loss term resulting from Darcy–Forchheimer’s law [19]. $∥{\vec{u}}_{b}∥$ is the mixture velocity magnitude, $D_{xx}$ and $D_{yy}$ are the inverse of the permeability, and $K_{xx}$ and $K_{yy}$ are the correction terms of Forchheimer. In this study, the first term of Equation (25) is neglected because the flow is turbulent. As the term has the same dimensions as a pressure gradient, Equation (25) is rewritten by:

(26) $$F_{t,x}=-\frac{\Delta P_{f,x}^{2\Phi }}{N_{R,x}P_{x}}\;\mathrm{and}\;F_{t,y}=-\frac{\Delta P_{f,y}^{2\Phi }}{N_{R,y}P_{y}}$$

$\Delta P_{f,i}^{2\Phi }$ is the two-phase frictional pressure drop, $N_{R,i}$ is the number of tube rows, and $P_{i}$ is the pitch in direction *i* $\left(\vec{x}\;\mathrm{or}\;\;\vec{y}\right)$ shown in Figure 2. From Equations (25) and 26, the unknown coefficients $K_{xx}$ and $K_{yy}$ are defined by:

(27) $$\left\{\begin{array}{cc} K_{xx} & =-\frac{\Delta P_{f,x}^{2\Phi }}{\frac{1}{2}\rho_{b}∥{\vec{u}}_{b}∥u_{b,x}N_{R,x}}\frac{1}{P_{x}}\\ K_{yy} & =-\frac{\Delta P_{f,y}^{2\Phi }}{\frac{1}{2}\rho_{b}∥{\vec{u}}_{b}∥u_{b,y}N_{R,y}}\frac{1}{P_{y}} \end{array}\right.$$

From the method developed by Consolini et al. [30] to define the two-phase frictional pressure drop over horizontal tube bundles, Equation (27) is reduced to:

(28) $$K_{xx}=-\frac{\lambda Eu}{P_{x}}{\left(\frac{P_{y}}{P_{y}-D_{ext}}\right)}^{2}\;\mathrm{and}\;K_{yy}=-\frac{\lambda Eu}{P_{y}}{\left(\frac{P_{x}}{P_{x}-D_{ext}}\right)}^{2}$$

where the Euler number, Eu, can be given by Zukauskas et al. [21]. Zukauskas et al. defined a correlation for the Euler number resulting from their experiments and experimental results from the literature. This law enables to determine frictional pressure drops for in-line and staggered tube bundles with $1.25\leq \;P/D_{ext}\;\leq 2.5$ and $10\leq \;Re\leq 10^{6}$. The Euler number is calculated as:

(29) $$Eu=k_{1}\sum_{i=0}^{4}\frac{c_{i}}{Re^{i}}$$

where $c_{i}$ and $k_{1}$ are coefficients given in reference [21]. The two-phase multiplier coefficient is written by Consolini et al. [30] as:

(30) $$\lambda =\Lambda +\left(1-\Lambda \right){\left(1-2x\right)}^{2}\;\mathrm{with}\;\Lambda ={\left(\frac{{\dot{m}}_{p}}{400}\right)}^{-1.5}$$

It is important to note that the two-phase multiplier factor is equal to 1 when the quality tends towards 0 (only liquid phase) and 1 (only gas phase). This key argument is not available with the correlation of Ishihara et al. [31] based on the Lockhart–Martinelli approach [32], which was the method used by Dowlati et al. in [26].

For complex or non-standard geometries, such as helicoidal tube bundles or corrugated tubes [33,34,35], there are no suitable or reliable correlations in the literature to compute the Forchheimer force $\vec{F_{t}}$. They can be obtained by experiments or numerical simulations by LES or RANS approaches but these methods imply a significant cost. In order to reduce computational time and cost, we suggest using a non-intrusive reduced model model (ROM). This parametric ROM simulates the flow in an REV (Representative Elementary Volume) of the tube bundle. The knowledge of this flow enables the Forchheimer term to be quickly obtained. In addition, the spatial distributions of the pressure and the velocity around each tube are precisely given by this approach. The ROM methodology is detailed in the next section.

## 3. Reduced Ordel Model on the REV to Compute the Forchheimer Term

### 3.1. Representative Elementary Volume

**Figure 3:**
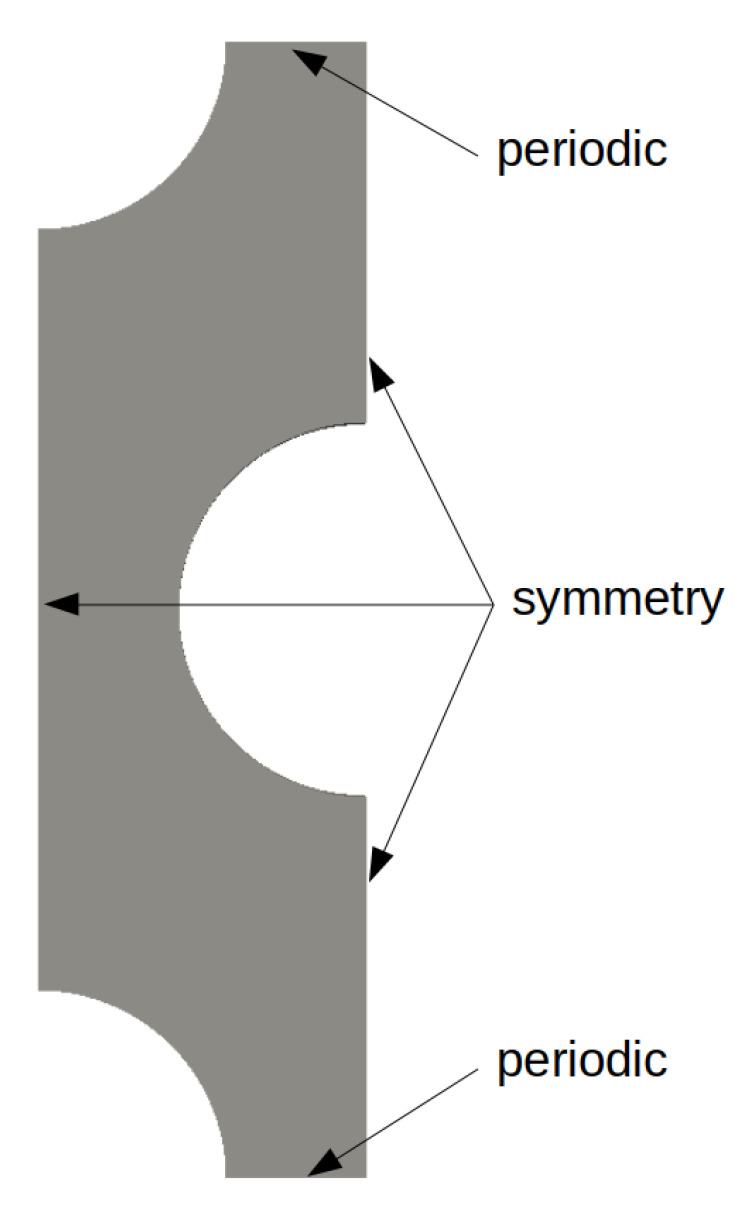
REV of the tube bundle of Dowlati et al. [26].

A Representative Elementary Volume (REV) of the tube bundle is considered in Figure 3, where the lower and upper boundary conditions are periodical. Governing equations for periodically fully developed flow are derived from the incompressible Navier–Stokes equations defined by:

(31) $$\left\{\begin{array}{c} \nabla \cdot \vec{u}=0\\ \frac{\partial \vec{u}}{\partial t}+\nabla \cdot \left(\vec{u}⊗\vec{u}\right)=-\frac{\nabla P}{\rho }+\nu \nabla^{2}\vec{u}+\frac{\vec{\beta }}{\rho } \end{array}\right.\;\;\mathrm{where}\;\;\vec{\beta }=\left(\begin{array}{c} 0\\ \beta \end{array}\right)$$

$\vec{u}$ is the periodic flow velocity:

(32) $$\vec{u}\left(x,y,t\right)=\vec{u}\left(x,y+2P_{y},t\right)$$

*P* represents the reduced pressure, which satisfies periodic boundary conditions,

(33) $$P\left(x,y,t\right)=P\left(x,y+2P_{y},t\right)$$

and the actual pressure is given by

(34) p(x,y,t)=−βy+P(x,y,t)

according to Patankar [36]. β is the linear component of the pressure, which is to be calculated iteratively for a fixed mass flow rate [37,38].

### 3.2. Definition of Reduced Bases

Model reduction techniques make it possible to quickly and inexpensively obtain the temporal dynamics of a complex flow. The principle of the reduced-order model (ROM) is to approximate the solution in a small dimension sub-vector space, which enables capturing the dominant characteristics of the physical phenomenon studied. The solution f(t,x) is written as a linear combination of a finite number of spatial basis functions $\Phi^{k}\left(x\right)$ as:

(35) $$f\left(t,x\right)\approx \sum_{k=1}^{q}a^{k}\left(t\right)\Phi^{k}\left(x\right)$$

*q* is relatively small compared to the problem size, and $a^{k}$ is the temporal coefficient. Here, *f* corresponds to the velocity $\vec{u}$ or the pressure *p*.

The most common method to compute the spatial basis Φ is the POD (Proper Orthogonal Decomposition) method [39,40]. However, the ability of the POD basis function to give the dynamic of the phenomenon studied is dependent of the information contained in the snapshots that form the basis. For instance, a POD basis built with snapshots for a Reynolds number $Re_{1}$ will not be able to predict the dynamics of the physical phenomenon for another Reynolds number $Re_{2}$. To increase the validity domain of the POD basis, it is possible to interpolate a set of POD bases $\Phi_{1},\cdots ,\Phi_{N}$ built for different values of Reynolds numbers $Re_{1},\cdots ,Re_{N}$ in order to obtain the basis associated to the desired Reynolds number. Standard interpolation techniques (RBF, Lagrange, Spline, etc.) are not very efficient and are not generally representative of the phenomenon studied. To get around this difficulty, a basis interpolation approach based on the results of differential geometry and, more particularly, on the properties of the Grassmann manifold can be used [25,41,42,43]. In this work, we consider the approach offered by Amsallem et al. [25] that is subsequently noted as ITSGM (Interpolation on the Tangent Subspace of the Grassmann Manifold). The algorithm is given in Algorithm A1.

Once the spatial basis for the desired parameter is defined, the temporal coefficients are usually computed by solving a system of differential equations (ROM) resulting from the Galerkin projection of the full model on the basis functions $\Phi^{k}\left(x\right)$ [44]. This method has the disadvantage of being costly and intrusive. Indeed, for each new value of Reynolds number, it is necessary to compute the coefficients of the ROM, which is costly. Moreover, derivative operators are difficult to assess using a commercial CFD code. Consequently, in this paper, we use the non-intrusive method Bi-CITSGM [23,45].

### 3.3. Description of the Bi-CITSGM Method

The methodology of the Bi-CITSGM is given in Algorithm A2 in the Appendix B. The first step of this method is to interpolate the spatial basis and the temporal basis, built by POD, using the ITSGM method. The singular value matrix is acquired by classical interpolation methods such as Lagrange, RBF, and Spline. Then, the ranking step aims to sort the interpolated spatial and time eigen modes with respect to the interpolated singular values. Indeed, as the spatial and temporal bases may not be in the same order as the singular values, orthogonal matrices need to be introduced. These calibration matrices are a solution to an optimization problem under constraints whose solution is analytically determined. For more details, see [23,24].

## 4. Validation of the Using of the Modified Mixture Model on the Dowlati’s Experiment

### 4.1. Study Configuration

**Figure 4:**
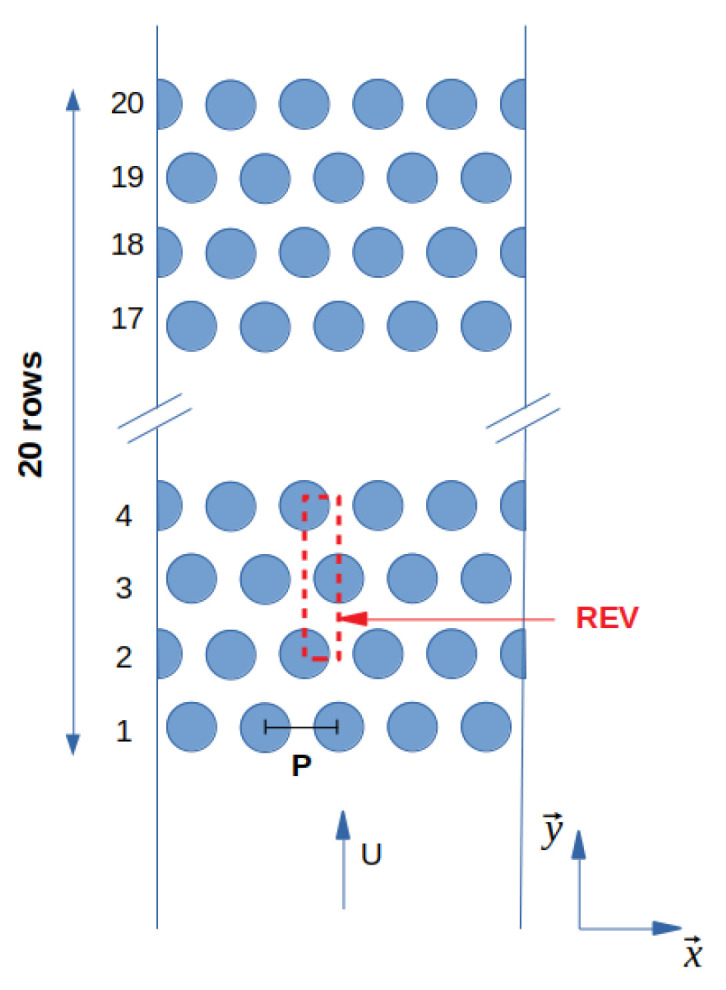
Staggered tube bundle from Dowlati’s experiment $\left(P/D_{ext}=1.75\right)$.

The methodology, presented in Section 2, was validated with the experiment of Dowlati et al. They made void fraction and friction pressure drop measurements for vertical two-phase flow of air–water across staggered in-line tube bundles with different pitch-to-diameter ratios. Here, the tube bundle, illustrated in Figure 4, is made up of 20 tube rows in a staggered arrangement with five tubes in each row and the ratio $P/D_{ext}$ is 1.75. Geometry dimensions are given in Table 1. The estimated uncertainties in the data done by Dowlati et al. are detailed in Table 2. The present study is done with quality range between $1.3\times 10^{-4}$ and $3\times 10^{-2}$ and mass flux range between 164 kg/(m${}^{2}\cdot$ s) and 538 kg/(m${}^{2}\cdot$ s).

**Figure 5:**
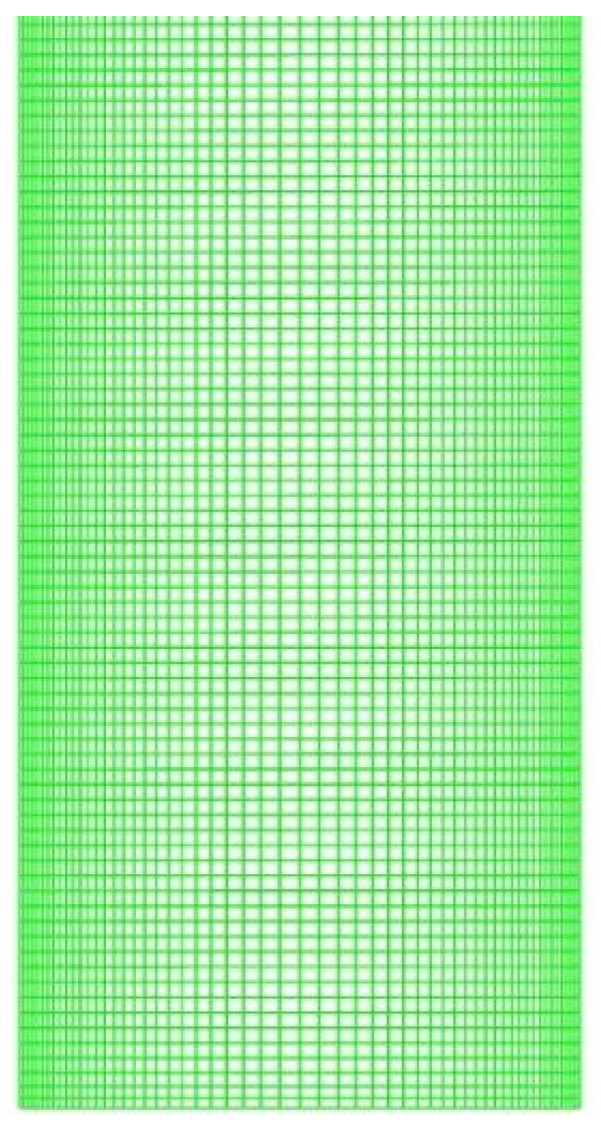
Mesh used for CFD calculations (size for the central cells = 3 mm/cell size along the border = 0.75 mm).

CFD calculations were performed with Ansys Fluent v2020R2. The tubes in the tube bundle were not represented. The tube bundle was modelled as a porous medium by adding a source term in the momentum equation. At the inlet, the homogeneous void fraction model is assumed. Thus, the homogeneous void fraction $\varepsilon_{H}$ and inlet phase velocities are written as follows,

(36) $${\vec{u}}_{g,in}={\left(\begin{array}{c} 0\\ \frac{x{\dot{m}}_{in}}{\rho_{g}\varepsilon_{H}} \end{array}\right)}_{\left(\vec{x},\vec{y}\right)}\;;\;{\vec{u}}_{l,in}={\left(\begin{array}{c} 0\\ \frac{\left(1-x\right){\dot{m}}_{in}}{\rho_{l}\left(1-\varepsilon_{H}\right)} \end{array}\right)}_{\left(\vec{x},\vec{y}\right)}$$

(37) $$\varepsilon_{H}=\frac{1}{1+\frac{\rho_{g}}{\rho_{l}}\frac{1-x}{x}}$$

where *x* is the quality. Calculations are initialized from the input boundary conditions. The mesh, illustrated in Figure 5, is defined in such a way as to ensure that the dimensionless wall distance $y^{+}$ is close to 1. The turbulent model k-ω SST [46] is used.

### 4.2. Influence of the Slip Model on the Void Fraction Prediction

In order to evaluate the influence of the slip model on the void fraction prediction, mean relative and absolute errors are introduced.

(38) $$E_{rel}=\frac{1}{N_{pt}}\sum_{i=1}^{N_{pt}}\frac{{\bar{\varepsilon }}_{i}^{calc}-{\bar{\varepsilon }}_{i}^{exp}}{{\bar{\varepsilon }}_{i}^{exp}}$$

(39) $$E_{abs}=\frac{1}{N_{pt}}\sum_{i=1}^{N_{pt}}\left({\bar{\varepsilon }}_{i}^{calc}-{\bar{\varepsilon }}_{i}^{exp}\right)$$

**Figure 6:**
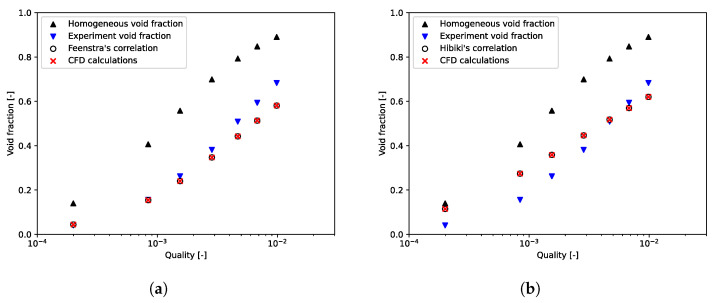
Void fraction results for air-water flow through Dowlati’s staggered tube bundle with ${\dot{m}}_{p}$ = 164 kg/(m${}^{2}$·s). (**a**) Feenstra’s correlation; (**b**) Hibiki’s correlation.

**Figure 7:**
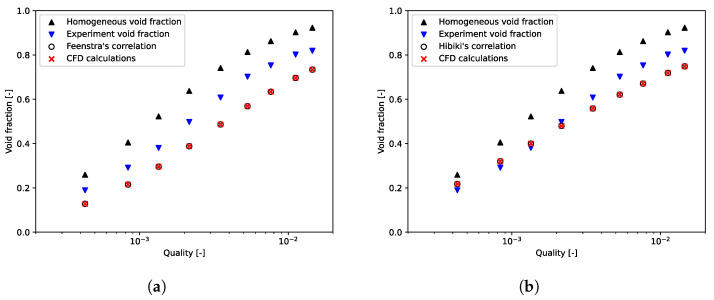
Void fraction results for air-water flow through Dowlati’s tube bundle with ${\dot{m}}_{p}$ = 401 kg/(m${}^{2}$·s). (**a**) Feenstra’s correlation; (**b**) Hibiki’s correlation.

**Figure 8:**
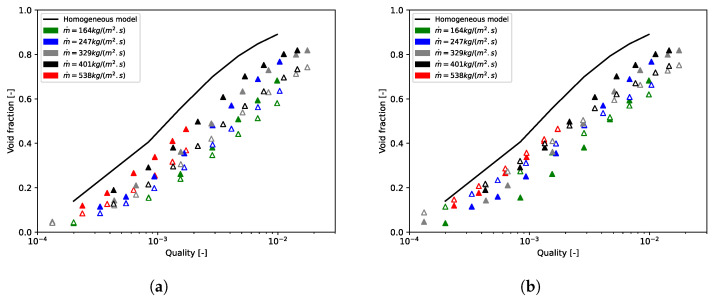
Void fraction as a function of quality and mass flux (▵ = results from CFD simulations and ▴ = experimental measurements). (**a**) Feenstra’s correlation; (**b**) Hibiki’s correlation.

$N_{pt}$, ${\bar{\varepsilon }}^{calc}$, and ${\bar{\varepsilon }}^{exp}$ are, respectively, the number of data and the computed and experimental void fractions averaged over the tube bundle. To prove that we need to apply a void fraction model to the mixture model, calculations were performed with the slip velocity formulation of Manninen. Here, the value of the void fraction postprocessed with this formulation always matches with the homogeneous void fraction. Figure 6 and Figure 7, for, respectively, ${\dot{m}}_{p}$ = 164 kg/(m${}^{2}$·s) and ${\dot{m}}_{p}$ = 401 kg/(m${}^{2}$·s), showed that the homogeneous void fraction, and thus the Manninen’s formulation, do not take into account the slip between phases for flows in tube bundle. Indeed, the relative error is about $E_{rel}=51.05\%$ and the absolute error $E_{abs}=0.15$. The results stress that it is important to implement a void fraction model appropriate for a two-phase cross-flow through a horizontal tube bundle. Moreover, for each mass flux, Figure 6 and Figure 7 demonstrate the correct implementation of Feenstra or Hibiki’s correlation in the CFD code. That is to say, the errors calculated subsequently in this paper derive only from the slip ratio model implemented and not from the numerical model. It can be seen in Figure 8, which depicts void fraction results as functions of quality and mass flux, that the void fraction increases with the quality and with the mass flux. Roser [47] justified this phenomenon by the upward movement of the gas phase against the liquid phase due to the buoyancy force making it all the more important that the mass flux is low. For higher mass flux, the gap with the homogeneous void fraction model is less significant. Indeed, the two phases are “well mixed” due to the increase in turbulence.

**Figure 9:**
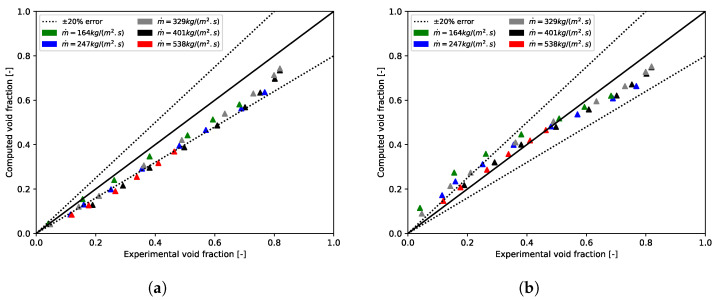
CFD computed void fraction versus experimental void fraction. (**a**) Feenstra’s correlation; (**b**) Hibiki’s correlation.

Figure 9 depicts CFD-computed void fractions versus experimental void fractions for all mass fluxes. Feenstra’s correlation always underpredicts the void fraction; however, errors are acceptable with $E_{rel}=17.49\%$ and $E_{abs}=0.07$. Errors seem to be more important for high mass fluxes and low qualities with some errors outside of range ±20%. Hibiki’s correlation overpredicts the results for ε≤0.5 and underpredicts it for ε>0.5. The relative error is a little higher than Feenstra’s correlation with $E_{rel}=21.81\%$, but the absolute error is better with $E_{abs}=0.05$. Some void fraction points are outside the range ±20% for low void fractions; however, this correlation is better for higher void fractions.

In order to improve the void fraction prediction, the Capillary number used in Feenstra’s correlation is now expressed as a function of upstream mass flux. The upstream mass flux and the mass flux between two tubes are linked by:

(40) $${\dot{m}}_{p}={\dot{m}}_{upstream}\frac{P}{P-D_{ext}}$$

**Figure 10:**
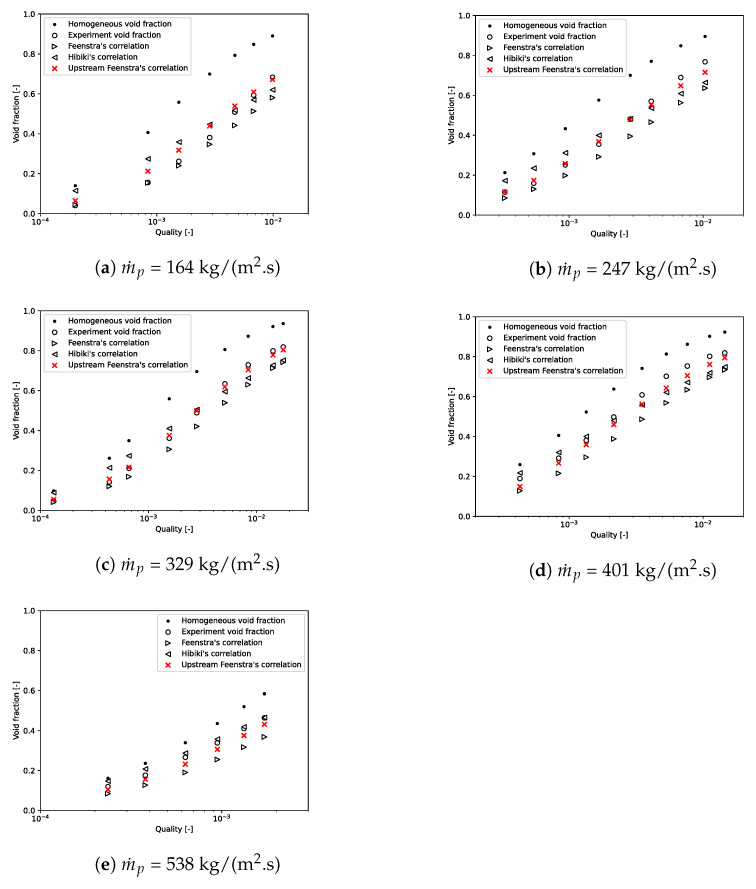
Comparison of void fraction results obtained by the different methods.

**Figure 11:**
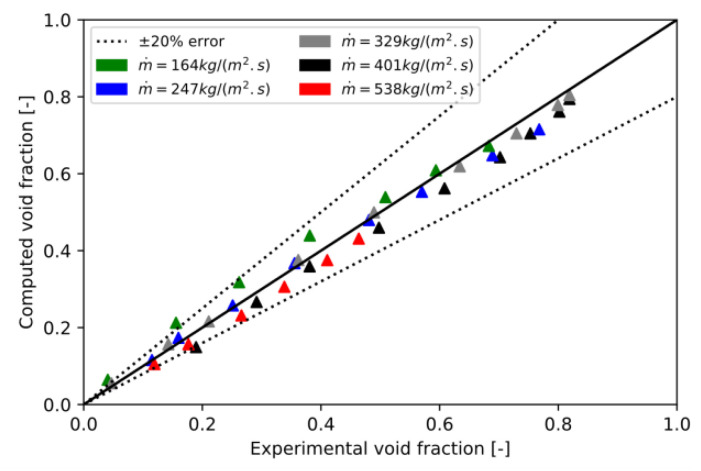
CFD computed void fraction with the modified Feenstra’s correlation versus experimental void fraction.

With this modified Feenstra’s correlation, named “Upstream Feenstra’s correlation”, the results were always improved compared to the experimental results, the original Feenstra’s correlation, and Hibiki’s correlation. Indeed, Figure 10 shows that the results with the modified Feenstra’s correlation were closer to experimental results than the original Feenstra’s correlation. It is important to note that only 10% of the simulations had a relative error for the void fraction higher than 20% for the modified Feenstra’s correlation. As can be seen in Figure 11, these points were located at low void fractions. For the higher void fraction, results were very close to the experiment. Compared to other correlations, this one has an absolute error of 0.03 and a relative error of 9.10%, which confirms the accuracy of the void fraction prediction.

**Figure 12:**
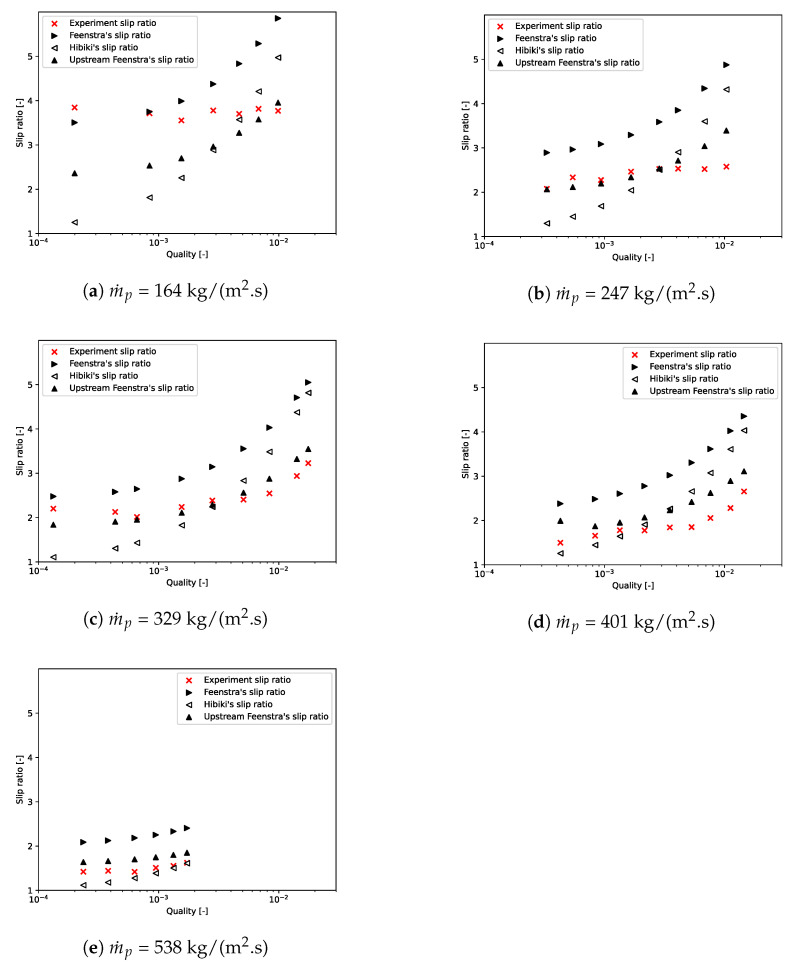
Comparison of slip ratio obtained by the different methods.

Figure 12 plots the slip ratio obtained by the experiment and Feenstra, Hibiki and upstream Feenstra’s correlations as a function of quality for each mass flux. As the void fraction increases with mass flux, it can be noticed that slip ratio decreases with the quality until reaching 1. For ${\dot{m}}_{p}\leq$ 247 kg/(m${}^{2}$·s), the slip ratio turns out to be relatively constant regardless of the quality. However, no correlation studied here captures this phenomenon. For ${\dot{m}}_{p}\geq$ 329 kg/(m${}^{2}$·s), the slip ratio increases with the quality, and correlations follow the same trend. Overall, the slip ratio obtained by the upstream Feenstra correlation is the closest to experimental results.

Now that we have verified the accurate prediction of the void fraction by rewriting the relative velocity and implementing a suitable void fraction model, we can focus on the pressure drop of the system. The porous media approach implies adding a source term to the momentum equation. We prove, in the next section, the possibility to use a non-intrusive parametric reduced model on an REV in order to compute this term.

## 5. Computation of the Pressure Drop in Tube Bundles by Using POD-ROM on an REV

The porous media approach involves the implementation of the Euler number (Equation (28)) in the Forchheimer’s correction term. Usually, this dimensionless number is computed with correlations coming from the literature. Here, we want to show that it is possible to compute this variable by a non-intrusive reduced-order model, Bi-CITSGM, applied to a REV of the tube bundle. The case of the tube bundle of Dowlati et al. is an case of application for which there is already a correlation resulting from the literature and for which the resolution of a reduced model is of little interest. However, we would like to extend this developed methodology to a case of a complex tube bundle for which there is no correlation from the literature. In addition, it is less expensive to define a correlation resulting from numerical calculations rather than from tests. First, the Bi-CITSGM method is validated on the REV, and then, the implementation on CFD simulations of the Dowlati’s experiment is discussed.

### 5.1. Validation of the Bi-CITSGM Method on the REV

Figure 3 depicts the 2-D REV of the tube bundle of Dowlati et al. with symmetric and periodic boundary conditions. The dimensions of transverse pitches and the tube diameter are given in Table 1. CFD calculations were done with OpenFOAM. The governing equations were unsteady and dimensionless, and the k-ω SST turbulence model is used. The flow is computed until the fully developed flow is established. The density of the fluid was always equal to 1 kg/m${}^{3}$, the desired mass flow was $4.7625\times 10^{-2}$ kg/s, and the viscosity varied according to the desired Reynolds number.

**Figure 13:**
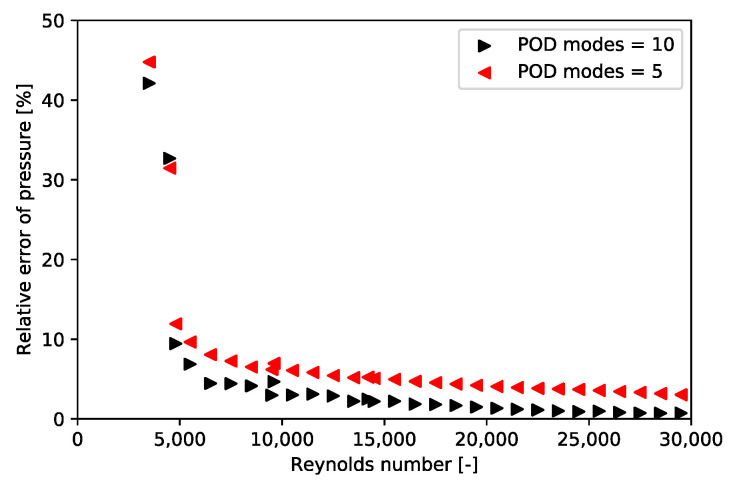
Relative error of the time- and area-weighted pressure average between Bi-CITSGM method and CFD calculation.

**Figure 14:**
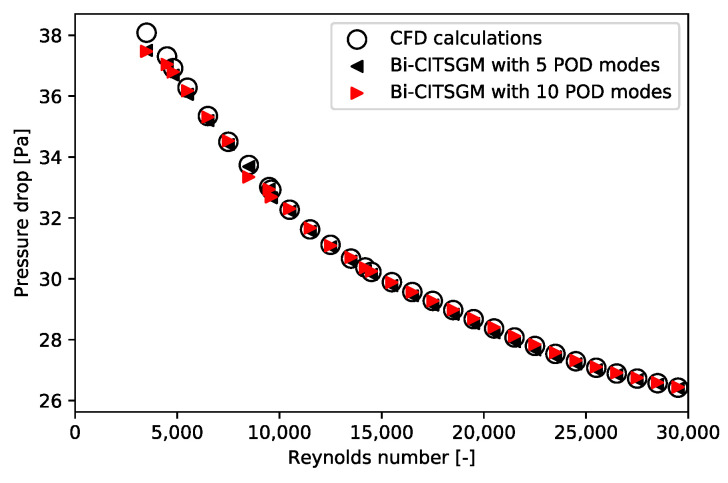
Time-weighted pressure drop average for Bi-CITSGM method and CFD calculations.

**Figure 15:**
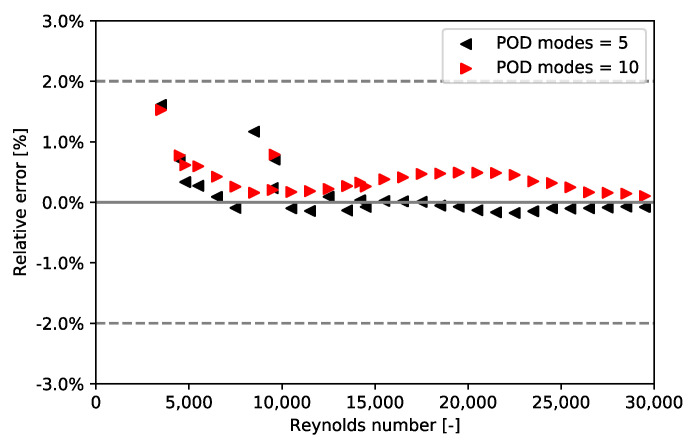
Relative error of the time weighted-pressure drop average between Bi-CITSGM method and CFD calculation.

**Figure 16:**
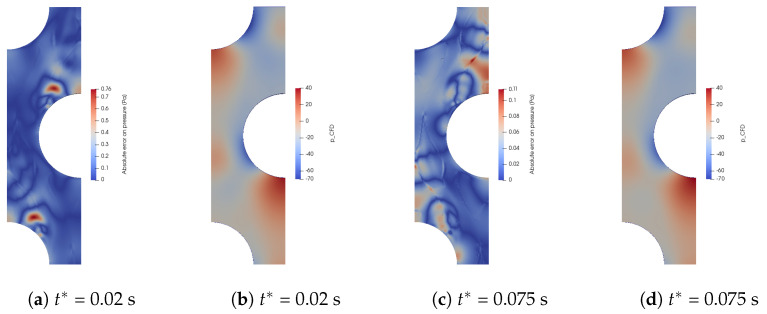
Absolute error between CFD calculations and Bi-CITSGM method for Re = 24,500.

All the considered training Reynolds numbers are {2000; 3000; …; 29,000; 30,000}. This range of training Reynolds numbers fits with the Reynolds number in each cell of the Dowlati’s simulation. For each Reynolds number, a thousand time steps are kept, and these snapshots are once and for all decomposed by POD. For each new Reynolds number, the Bi-CITSGM method gives the results almost instantly. In Figure 13, Figure 14 and Figure 15, solving the ROM with five or ten POD modes are compared to the reference CFD calculation for each Reynolds number. The Bi-CITSGM method enables determining the pressure field with an accuracy less than 10%, except for Reynolds numbers less than 5000 (Figure 13). To increase the accuracy of pressure fields, the spacing between the training Reynolds numbers should be reduced in this range. However, the key parameter in this section is the pressure drop. Figure 14 and Figure 15 show that the pressure drop prediction is much less than 10% even for Reynolds numbers less than 5000. For instance, at a Reynolds number of 24,500, the absolute error between the CFD reference pressure field and the interpolated pressure field at two given times is represented in Figure 16. The highest errors are rather local in the REV, and overall, the interpolated pressure field is very close to the CFD reference. Following the good results achieved by the Bi-CITSGM method on the REV, the results in the next subsection are plotted by keeping only five POD modes.

### 5.2. Validation of the Implementation of the Bi-CITSGM Method on the Dowlati’s Experiment

**Figure 17:**
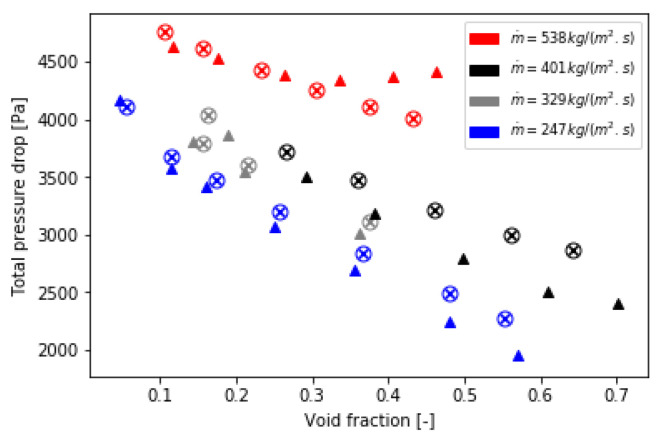
Total pressure drop of Dowlati’s tube bundle (×: *Zukauskas*; ∘: *Bi-CITSGM*; ▴: *experiment*).

**Figure 18:**
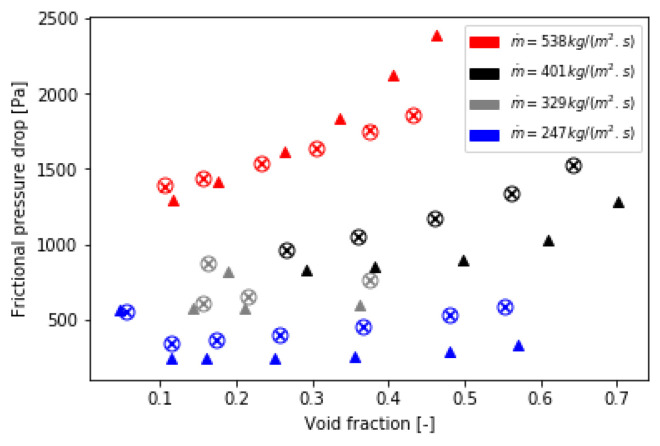
Frictional pressure drop of Dowlati’s tube bundle (×: *Zukauskas*; ∘: *Bi-CITSGM*; ▴: *experiment*).

The Bi-CITSGM method is solved at each REV of the tube bundle and each iteration in order to compute the Forchheimer term. In addition, the void fraction model of Feenstra et al. is always implemented. Figure 17 compares the total two-phase pressure drop of Dowlati’s tube bundle given by the experiment, the Zukauskas correlation implementation and the Bi-CITSGM method implementation in the Forchheimer term. Total pressure drops well fit with the experiment results at low void fractions. Errors become higher when the void fraction increases; however, the post-processing of the pressure drop from the paper of Dowlati et al. [26] is not immediate, nor is it very accurate. The post-process of the pressure drops resulting from the implementation of the Zukauskas correlation and that of the Bi-CITSGM method in the source term are superimposed on the graph. These results validate the suggested approach. Likewise, in Figure 18, the two-phase frictional pressure drops are plotted for different mass flux and are compared to the experiment results. We note that the two-phase frictional pressure drop is highly dependent on the mass flux and less on the void fraction. On the contrary, the gravitational pressure drop decreases when the void fraction increases and is barely dependent on the mass flux. These results are consistent with the physical phenomena that occur in a two-phase flow through a tube bundle. Consequently, the aim to determine the momentum sink by the non-intrusive reduced-order model, Bi-CITSGM, is validated by the results presented in this subsection.

## 6. Conclusions

In order to predict the thermal–hydraulic performance of an adiabatic upward air-water flow through a horizontal tube bundle, two approaches are suggested. They are validated with the experimental results of Dowlati et al. First, the prediction of the void fraction on the tube bundle was improved by using the mixture model and rewriting the drift velocity as a function of slip. Two correlations coming from the literature, Hibiki et al. [17] and Feenstra et al. [16], are compared to the experimental results. They are given similar results with a relative error about 20%. Moreover, we showed that the definition of the Capillary number with the upstream mass flux in Feenstra’s correlation significantly improves the void fraction prediction with a relative error under 10%. Second, the CFD porous media approach used implies adding a momentum sink to the governing momentum equation named the Darcy–Forchheimer term. Usually, pressure drop correlations coming from the literature have been used to compute the Forchheimer term except for complex and non-usual geometry for which there is no correlation. In this instance, we demonstrate that it is possible to determine a numerical pressure drop correlation by solving a non-intrusive parametric reduced-order model of the flow through a Representative Elementary Volume of the tube bundle. In the case of the straight tube bundle of Dowlati et al., the Bi-CITSGM method is consistent with the Zukauskas correlation [21]. Moreover, there is a short gap with the experimental results despite a significant possible post-processing error. The two proposed methods that yield satisfactory results need to be expanded. For instance, it would be interesting to simulate a two-phase parallel-flow in a staggered vertical tube bundle with the mixture model modified by the rewriting of the drift velocity. Moreover, the use of a non-intrusive reduced-order model applied to a non-usual geometry of REV in order to compute the Forchheimer term could be an axis of development.

## Figures and Tables

**Table 1:** Dimensions of the tube bundle.

	Value
Outer diameter of a tube $D_{ext}$ [mm]	12.7
Ratio $P/D_{ext}$ [-]	1.75
Pitch $P_{x}$ [mm]	22.225
Pitch $P_{y}$ [mm]	19.25
Width $L=5P_{x}$ [mm]	111.125
Height $H=20P_{y}$ [mm]	385

**Table 2:** Estimated uncertainties in the data given by Dowlati et al. [26].

Parameter	Uncertainty
Quality	±2%
Void fraction	±0.05
Mass flux	±2%
Frictional two-phase pressure drop	±15%

## Data Availability

Not applicable.

## Abbreviations

    The following abbreviations are used in this manuscript:

CFD	Computational Fluid Dynamic
POD	Proper Orthogonal Decomposition
ROM	Reduced-order model
REV	Representative Elementary Volume
ITSGM	Interpolation on the Tangent Subspace of the Grassmann Manifold
Bi-CITSGM	Bi Calibrated Interpolation on the Tangent Subspace of the Grassmann Manifold
RBF	Radial Basis Function
IDW	Inverse Distance Weighting

## Appendix A. Algorithm of the ITSGM Method

Let $\Phi_{{}_{\gamma_{1}}},\Phi_{{}_{\gamma_{2}}},\cdots ,\Phi_{{}_{\gamma_{N_{p}}}}$ be a set of POD basis and $\left[\Phi_{{}_{\gamma_{1}}}\right],\left[\Phi_{{}_{\gamma_{2}}}\right],\cdots ,\left[\Phi_{{}_{\gamma_{N_{p}}}}\right]$ the sub-vector space associated belonging to the Grassmann manifold. By using the definitions of the geodesic path, exponential application, and logarithm application, the aim is to approximate the subspace $\left[\Phi_{{}_{\tilde{\gamma }}}\right]$ amount for a new parameter $\tilde{\gamma }\neq \gamma_{i}$. The different steps of the ITSGM method come hereafter.

**Algorithm A1** ITSGM Algorithm.
(a) Choose a reference point $\left[\Phi_{{}_{\gamma_{i_{0}}}}\right]$ where $i_{0}\in \left\{1,\cdots ,N_{p}\right\}$. (b) For $i\in \{1,\cdots ,N_{p}\}$, determine the vector $X_{{}_{i}}={\mathrm{Log}}_{{}_{\left[\Phi_{{}_{\gamma_{i_{0}}}}\right]}}\left(\Phi_{{}_{\gamma_{i}}}\right)$ with (A1) $$X_{{}_{i}}=U_{i}\arctan\left(\Sigma_{i}\right)V_{i}^{T},i=1,\cdots ,N_{p}$$ where $U_{i}\Sigma_{i}V_{i}^{T}$ is the truncated SVD decomposition of $\left(I-\Phi_{{}_{\gamma_{i_{0}}}}{\Phi_{{}_{\gamma_{i_{0}}}}}^{T}\right)\Phi_{{}_{\gamma_{i}}}{\left({\Phi_{{}_{\gamma_{i_{0}}}}}^{T}\Phi_{{}_{\gamma_{i}}}\right)}^{-1}$. (c) Interpolate $X_{{}_{1}},X_{{}_{2}},\cdots ,X_{{}_{N_{p}}}$ and obtain the initial velocity $X_{{}_{\tilde{\gamma }}}$ linked with the new parameter $\tilde{\gamma }$. As the tangent space $T_{{}_{\left[{\Phi_{{}_{i_{0}}}}_{}\right]}}G\left(q,N_{}\right)$ is a vector space, the interpolation standard technique can be used like Lagrange or RBF. (d) Determine the interpolate sub-vector space (A2) $$\Phi_{{}_{\tilde{\gamma }}}=\Phi_{{}_{\gamma_{i_{0}}}}\tilde{V}\cos\left(\tilde{\Sigma }\right)+\tilde{U}\sin\left(\tilde{\Sigma }\right)$$ where $\tilde{U}\tilde{\Sigma }{\tilde{V}}^{T}$ is the SVD decomposition truncated of $X_{{}_{\tilde{\gamma }}}$.

## Appendix B. Algorithm of the Bi-CITSGM Method

Let the POD decomposition of order *q* of the matrices $S_{{}_{\gamma_{i}}}$ linked to the parameter $\gamma_{i}$ such as

$$S_{{}_{\gamma_{i}}}\approx U_{\gamma_{i}}\Sigma_{{}_{\gamma_{i}}}V_{\gamma_{i}}^{T}$$

where $U_{\gamma_{i}}\in R^{N_{x}\times q}$ et $V_{\gamma_{i}}\in R^{N_{s}\times q}$ are, respectively, the spatial and temporal bases, and $\Sigma_{{}_{\gamma_{i}}}\in R^{q\times q}$ is the singular value matrix. Obtaining the solution $S_{{}_{\tilde{\gamma }}}$ for a new parameter, $\tilde{\gamma }\neq \gamma_{i}$ is given by the online step of the Bi-CITSGM method defined below.

**Algorithm A2** Bi-CITSGM Algorithm.
*Offline step (do this step only once):* (a) For $i=1,\cdots ,N_{p}$, to approximate the snapshot matrices $S_{{}_{\gamma_{i}}}$, use the truncated SVD decomposition of order *q* as follows: (A3) $$S_{{}_{\gamma_{i}}}\approx U_{\gamma_{i}}\Sigma_{{}_{\gamma_{i}}}V_{\gamma_{i}}^{T}$$ where $U_{\gamma_{i}}\in R^{N_{x}\times q}$ and $V_{\gamma_{i}}\in R^{N_{s}\times q}$ are, respectively, the spatial and time basis and $\Sigma_{{}_{\gamma_{i}}}\in R^{q\times q}$ is the singular values matrix. *Online step:* (b) Interpolate the singular values matrix $\Sigma_{{}_{\gamma_{i}}}$ with the classical interpolation method (Lagrange, RBF, etc.) and build the matrix $\tilde{\Sigma }$ for the new parameter $\tilde{\gamma }$. (c) Approximate the spatial basis $U_{\tilde{\gamma }}$ and the temporal basis $V_{\tilde{\gamma }}$ with the reduced basis interpolation method ITSGM (described in the Algorithm A1). (d) Adjust the signs of the modes of the training basis by multiplying the *j*th spatial mode ${\Phi_{{}_{\gamma_{k}}}}^{j}$ and temporal mode ${\Lambda_{{}_{\gamma_{k}}}}^{j}$ by −1 if the following condition is met: $\left|\right|{\Phi_{{}_{\gamma_{k_{0}}}}}^{j}-{\Phi_{{}_{\gamma_{k}}}}^{j}{\left|\right|}_{2}>||{\Phi_{{}_{\gamma_{k_{0}}}}}^{j}+{\Phi_{{}_{\gamma_{k}}}}^{j}{\left|\right|}_{2}$ with $k_{0}=\underset{i\in \{1,\cdots ,N_{p}\}}{\mathrm{argmin}}\;\;{\mathrm{dist}}_{G}\left(\Phi_{{}_{\tilde{\gamma }}},\Phi_{{}_{\gamma_{i}}}\right)$. (e) Calculate the considered coefficients $\omega_{i}$ and $\kappa_{i}$ (A4) $$\omega_{i}=\frac{{\mathrm{dist}}_{G}{\left(U_{\tilde{\gamma }},U_{\gamma_{i}}\right)}^{-m}}{\overset{N_{p}}{\sum_{k=1}}{\mathrm{dist}}_{G}{\left(U_{\tilde{\gamma }},U_{\gamma_{k}}\right)}^{-m}}\;\kappa_{i}=\frac{{\mathrm{dist}}_{G}{\left(V_{\tilde{\gamma }},V_{\gamma_{i}}\right)}^{-l}}{\overset{N_{p}}{\sum_{k=1}}{\mathrm{dist}}_{G}{\left(V_{\tilde{\gamma }},V_{\gamma_{k}}\right)}^{-l}}$$ (f) Calculate λ the diagonal matrix of eigenvalues, and *P* the matrix of eigenvectors by verifying the following eigenvalue decomposition: (A5) $$\sum_{i=0}^{N_{p}}\sum_{j=0}^{N_{p}}\omega_{i}\omega_{j}U_{\gamma_{i}}^{T}U_{\tilde{\gamma }}U_{\tilde{\gamma }}^{T}U_{\gamma_{j}}=P\lambda P^{T}$$ (g) Calculate η the diagonal matrix of eigenvalues, and *H* the matrix of eigenvectors by verifying the following eigenvalue decomposition: (A6) $$\sum_{i=0}^{N_{p}}\sum_{i=0}^{N_{p}}\kappa_{i}\kappa_{j}V_{\gamma_{i}}^{T}V_{\tilde{\gamma }}V_{\tilde{\gamma }}^{T}V_{\gamma_{j}}=H\eta H^{T}$$ (h) Calculate the orthogonal matrices *K* and *Q* given by: (A7) $$K=U_{\tilde{\gamma }}\left(\sum_{i=0}^{N_{p}}\omega_{i}U_{\gamma_{i}}\right)P\lambda^{-\frac{1}{2}}P^{T}$$ (A8) $$Q=V_{\tilde{\gamma }}\left(\sum_{i=0}^{N_{p}}\kappa_{i}V_{\gamma_{i}}\right)H\eta^{-\frac{1}{2}}H^{T}$$ (i) Build the interpolate snapshot matrix defined by: (A9) $$S_{{}_{\tilde{\gamma }}}=U_{\tilde{\gamma }}K\tilde{\Sigma }Q^{T}V_{\tilde{\gamma }}^{T}$$
